# Modeling a potential SANS countermeasure by experimental manipulation of the translaminar pressure difference in mice

**DOI:** 10.1038/s41526-020-00109-5

**Published:** 2020-07-31

**Authors:** Guofu Shen, Schuyler S. Link, Xiaofeng Tao, Benjamin J. Frankfort

**Affiliations:** 1 Department of Ophthalmology, Baylor College of Medicine, Houston, TX USA; 2 Department of Neuroscience, Baylor College of Medicine, Houston, TX USA; 3 Center for Space Medicine, Baylor College of Medicine, Houston, TX USA

**Keywords:** Optic nerve diseases, Brain injuries, Physiology, Anatomy

## Abstract

The spaceflight-associated neuro-ocular syndrome (SANS), which may present after prolonged exposure to microgravity, is thought to occur due to elevated intracranial pressure (ICP). Intracranial pressure interacts with intraocular pressure (IOP) to define the translaminar pressure difference (TLPD; IOP−ICP). We combined inducible models of ICP and IOP elevation in mice to interrogate the relationships among ICP, IOP, and TLPD, and to determine if IOP elevation could mitigate the phenotypes typically caused by elevated ICP and thereby serve as a countermeasure for SANS. Ten C57BL6J mice of both genders underwent experimental elevation of ICP via infusion of artificial cerebrospinal fluid into the subarachnoid space. One eye also underwent experimental elevation of IOP using the bead injection model. Intraocular pressure and ICP were monitored for 2 weeks. Optokinetic-based contrast sensitivity was measured at baseline and after 2 weeks, and post-mortem studies of optic nerve and retina anatomy were performed. Photopic contrast sensitivity was reduced more in IOP elevated than control eyes. Scotopic contrast sensitivity was reduced similarly in IOP elevated and control eyes. However, the pattern of scotopic vision loss was not uniform in IOP elevated eyes; there was minimal loss in eyes that most closely approximated the normal TLPD. Optic nerve axon loss, increased optic nerve disorganization, and retinal ganglion cell loss all occurred similarly between IOP elevated and control eyes. Elevation of IOP in eyes with elevated ICP may counterbalance some effects on vision loss but exacerbate others, suggesting complex relationships among IOP, ICP, and TLPD.

## Introduction

Retinal ganglion cells (RGCs) are the obligate output neuron of the mammalian retina and their axons are the primary constituents of the optic nerve. The optic nerve exits the eye posteriorly and travels to the brain where RGC axons synapse on higher order neurons. Along this path, two primary forces influence RGC axons: the intraocular pressure (IOP) inside the eye and at the anterior optic nerve head, and the intracranial pressure (ICP) at the posterior optic nerve head and along the course of the optic nerve^1–4^. Idiopathic or secondary increases in either ICP or IOP result in vision-threatening diseases such as spaceflight-associated neuro-ocular syndrome (SANS; ICP), idiopathic intracranial hypertension (IIH; ICP), and glaucoma (IOP)^5–7^.

The pressure difference between IOP and ICP defines the translaminar pressure difference (TLPD, or IOP−ICP). The TLPD is likely of critical importance to optic nerve health and may help explain the pathology of certain optic nerve diseases. One example, SANS, occurs when ICP is elevated but IOP is normal and the TLPD is reduced, and results in optic disc swelling and a variety of changes in vision which may be asymmetric^6,8,9^. Another example, normal tension glaucoma, occurs when IOP is again normal but ICP is reduced and the TLPD is increased, and results in optic nerve cupping and classic changes in vision^1–3,10–13^. Not surprisingly, several groups have suggested that IOP and ICP exist in a delicate balance, and that the manipulation of one pressure can counterbalance abnormalities in the other^14–20^. Unfortunately, the relative inaccessibility of directly measured ICP in humans, which requires either lumbar puncture or intracranial instrumentation, hinders the direct testing of these hypotheses, especially under conditions of microgravity^21^. Indirect, noninvasive measurements of ICP exist, but have limitations^22–25^. Thus, because of the difficulty surrounding measurements of ICP in humans and even model systems, controlled experimental data about the impact of ICP on the TLPD and optic nerve function and biomechanics are limited^26–30^. In the context of exposure to prolonged microgravity, data are even more limited given the small number of subjects available for study and the possibility of unclarified effects of microgravity on the mechanisms of ICP and IOP homeostasis^31,32^. Theoretically, correction of ICP, IOP, or TLPD imbalances by manipulation of one or both pressures could alleviate some disease symptoms.

We developed a technique in mice to allow for the elevation and measurement of ICP in living, active animals via an infusion system linked to a pressure sensor in the sub-arachnoid space^33^. With this approach, we previously confirmed that ICP elevation affects the visual pathways, RGCs, and the optic nerve in predictable ways, and may therefore serve as a model for SANS and other terrestrial diseases of elevated ICP^33–35^. In contrast to experimental systems in mice to increase ICP, experimental systems to increase IOP in living mice are well-established and abundant^36–40^. We have previously used one model, the bead injection model, to probe various components of the visual system as a model for glaucoma^37,41–43^. In this manuscript, we combine techniques to manipulate IOP and ICP simultaneously in living, active mice, and test the hypothesis that elevated IOP can counterbalance the effects of elevated ICP. In mice with experimentally elevated ICP to simulate SANS, the IOP of one eye was secondarily increased by bead injection, while the other eye received saline injection as an internal control. Intracranial pressure was increased to a higher level than was IOP. We found that both ICP and IOP−ICP relationships were important factors in determining vision loss after concomitant IOP and ICP elevation, while IOP alone was not. Increased ICP and IOP acted additively to reduce photopic contrast sensitivity across a range of negative TLPDs. Scotopic contrast sensitivity loss was similarly exacerbated, but only among eyes with large negative deviations of TLPD from baseline, and was actually mitigated in eyes with smaller deviations of TLPD from baseline. The magnitude and quality of RGC and axonal losses were similar among all eyes. These results indicate a complex relationship between ICP and IOP that can impact vision in a number of potentially unpredictable ways.

## Results

### Elevation of ICP and IOP

**Fig. 1:**
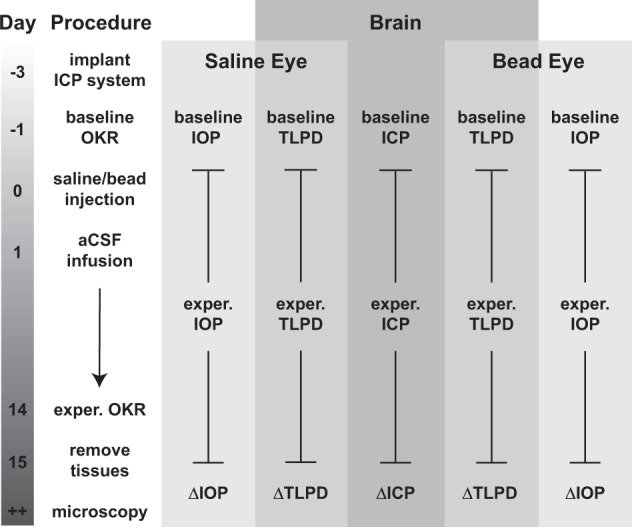
Experimental schematic. To begin the experiment, surgery was performed to implant the infusion cannula and pressure-monitoring probe for future ICP elevation and measurement. Once animals had recovered, baseline measurements of IOP and ICP were taken, and the baseline TLPD was calculated. On the same day, the baseline OKR to measure contrast sensitivity was conducted. The next day, mice received saline injection in one eye (IOP control) and bead injection in the other eye (IOP elevation). The following day, ICP was raised via infusion of aCSF into the subarachnoid space. This infusion was maintained for the duration of the experiment. Beginning with the day of ICP elevation (the day after IOP elevation), experimental ICP and IOP were measured at defined intervals over the next 14 days and used to calculate the experimental TLPD. After 14 days, the experimental OKR to re-measure contrast sensitivity was conducted. The day after, animals were killed and their eyes and optic nerves collected for retinal immunofluorescence and transmission electron microscopy, respectively. The differences between the experimental and baseline values of ICP, IOP, and TLPD were used to calculate ΔICP, ΔIOP, and ΔTLPD.

**Fig. 2:**
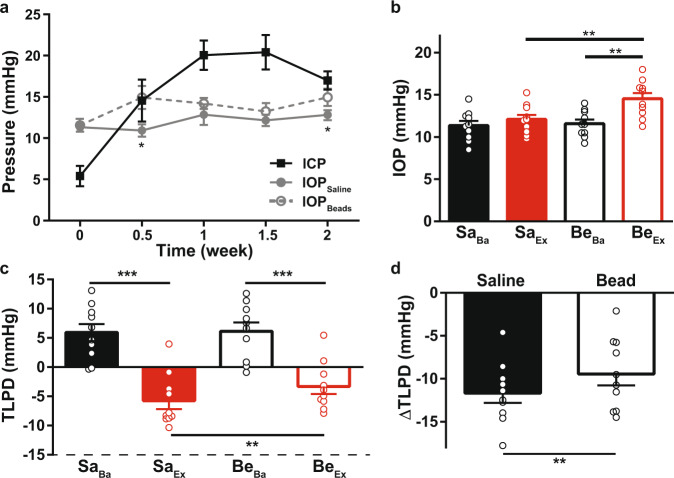
Changes in IOP, ICP, and TLPD. **a** Combined plot of mean baseline (time = 0) and experimental (all other time points) IOP and ICP values. ICP, IOP elevation (IOP_Beads_; gray open circle connected with dotted line) and contralateral control (IOP_Saline_; gray solid circle connected with a solid line) are shown. **b** Mean baseline IOP (black bars; Sa_Ba_ = saline baseline; Be_Ba_ = beads baseline) and mean experimental IOP (average of all post-injection values, per eye; red bars; Sa_Ex_ = saline experimental; Be_Ex_ = beads experimental). **c** Mean baseline and experimental values for TLPD. Baseline TLPDs are in black and experimental TLPDs are in red. Experimental values of TLPD are different from baseline values (Sa_Ba_ vs Sa_Ex_ and Be_Ba_ vs Be_Ex_; both <0.001). **d** ΔTLPD for saline- and bead-injected animals. ΔTLPD represents the change in TLPD from baseline (experimental − baseline). For all panels, *N* = 10 and error bars = 1 SEM. *p < 0.05, **p < 0.01, ***p < 0.001.

C57BL6J mice of both genders (*n* = 10; 6 female and 4 male) underwent experimental elevation of ICP via continuous infusion of artificial CSF (Fig. 1). Intracranial pressure elevation was maintained for 2 weeks and ICP increased from an average baseline level of 5.44 ± 1.24 mmHg to an average experimental level of 18.02 ± 1.40 (*p* < 0.001; Fig. 2a). Each animal also received bead injection in one eye and saline injection in the other eye. The elevation of IOP in only one eye allowed us to test if elevation of IOP was sufficient to mitigate phenotypes caused by elevated ICP, and to compare to contralateral control eyes of the same animal. For bead-injected eyes, the average baseline IOP was 11.58 ± 0.49 mmHg and the average experimental IOP was 14.53 ± 0.68 (*p* < 0.01; Fig. 2a, b). For saline-injected eyes, the average baseline IOP was 11.35 ± 0.56 mmHg and the average experimental IOP was 12.08 ± 0.54 (*p* = 0.22; Fig. 2a, b). Over the course of the experiment, bead-injected eyes had a higher IOP than saline-injected eyes (ANOVA with repeated measures, *p* = 0.023), but not at every time point (Fig. 2a, asterisks). The average experimental IOP after bead injection was higher than both baseline IOP prior to bead injection and experimental IOP after saline injection (*p* = 0.006 and *p* = 0.004, respectively, Fig. 2b). Baseline IOP and ICP values were used to calculate the baseline TLPD (IOP−ICP), which was positive, as expected, since IOP is typically higher than ICP (Fig. 2c). As anticipated, the experimental TLPD was negative, since the magnitude of ICP elevation was greater than the magnitude of IOP elevation (Fig. 2c). This also created an average negative value of ΔTLPD, the difference between the experimental and baseline TLPD (Fig. 2d; see “Methods”). ΔTLPD was more negative for saline-injected eyes (−11.67 ± 1.14 mmHg) than for bead-injected eyes (−9.44 ± 1.32 mmHg; *p* = 0.008; Fig. 2d). While mice do not have a collagenous lamina cribrosa, they do have an astrocytic lamina, and therefore the term TLPD is used throughout the manuscript^44^.

### Changes in contrast sensitivity

**Fig. 3:**
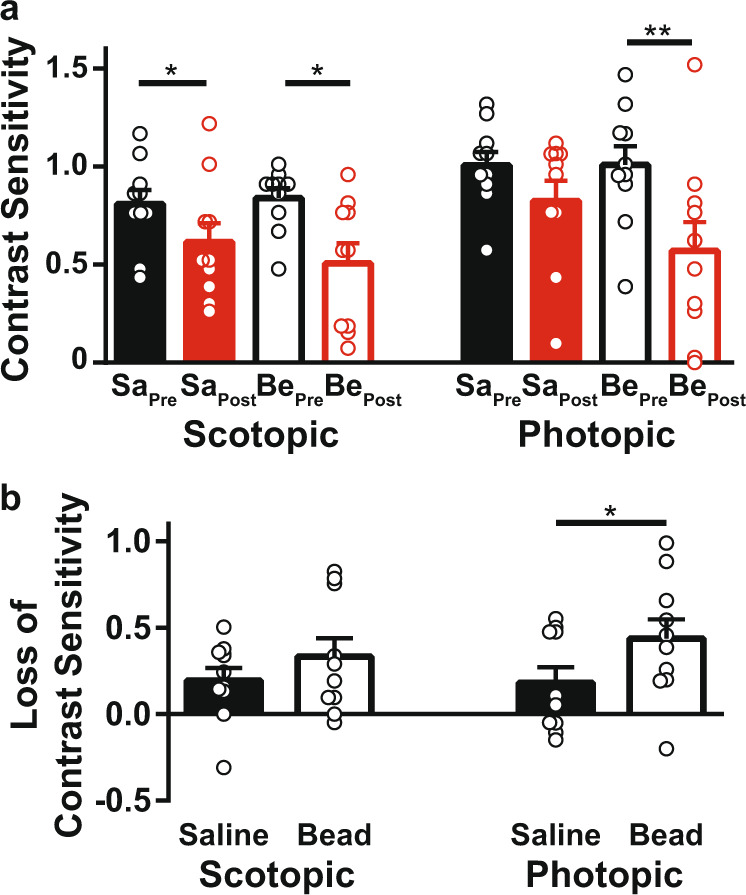
Reduced contrast sensitivity after ICP and IOP elevation. **a** Scotopic (left) and photopic (right) contrast sensitivity was measured simultaneously from saline-injected (filled bars) and bead-injected (open bars) eyes. The pre-elevation measurements (saline = Sa_Pre_ and beads = Be_Pre_; black) and the post-experiment measurements (saline = Sa_Post_ and beads = Be_Post_; red) are both shown. **b** Contrast sensitivity, recalculated as a loss over time in the same eye and replotted. For all panels, *N* = 10 and error bars = 1 SEM. *p < 0.05. **p < 0.01.

Scotopic and photopic contrast sensitivity were measured using an optokinetic technique in both eyes of all animals. These measurements were taken at the start of the experiment (after surgery to implant the ICP manipulation machinery but prior to any IOP or ICP elevation) and at the end of the 2-week experiment. Regardless of whether eyes received bead or saline injection, there was a reduction of contrast sensitivity over time (scotopic saline-injected eyes, *p* = 0.026; scotopic bead-injected eyes, *p* = 0.012; photopic saline-injected eyes, *p* = 0.077; photopic bead-injected eyes, *p* = 0.0036; Fig. 3a). Bead-injected eyes lost more contrast sensitivity than saline-injected eyes under only photopic conditions (paired *t* test, *p* = 0.24 for scotopic and 0.019 for photopic; Fig. 3b). Thus, there did not appear to be a protective effect of IOP elevation on ICP-related vision loss. Rather IOP elevation in the setting of ICP elevation incurred additional losses of photopic contrast sensitivity loss but produced no global impact on scotopic contrast sensitivity.

**Fig. 4:**
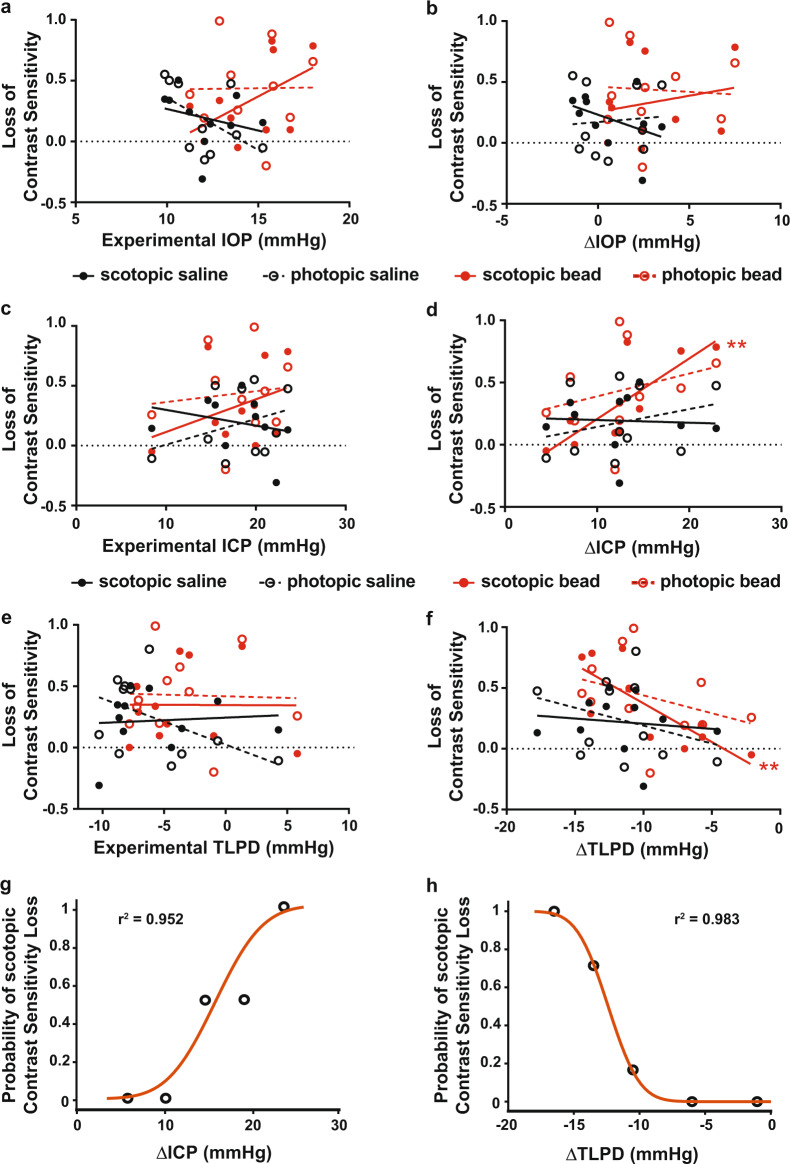
Magnitude effects of IOP, ICP, and TLPD on contrast sensitivity. Losses in contrast sensitivity were plotted against experimental IOP (**a**), ΔIOP (**b**), experimental ICP (**c**), ΔICP (**d**), experimental TLPD (**e**) and ΔTLPD (**f**). **a**−**f** The magnitude of contrast sensitivity loss for saline-injected (black) and bead-injected (red) eyes under scotopic (solid circle, solid trend line) and photopic (open circle, dotted trend line) conditions is shown. There is a strong relationship between both ΔICP and ΔTLPD and the magnitude of scotopic contrast sensitivity loss, but only for bead-injected eyes. Logistic regression curve plotting the probability of scotopic contrast sensitivity loss according to ΔICP and ΔTLPD (**g**, **h**). **p < 0.01.

To further explore the effect of the TLPD on contrast sensitivity, we assessed the individual impacts of the magnitude of experimental IOP, experimental ICP, ΔIOP, and ΔICP on contrast sensitivity loss. Interestingly, we did not detect any correlation between experimental IOP or ΔIOP and scotopic or photopic contrast sensitivity loss, regardless of whether an eye received bead or saline injection (Fig. 4a, b, Table 1). This suggested that IOP level alone did not impact the degree of contrast sensitivity loss. In contradistinction, we did detect an effect of ΔICP, but not experimental ICP, on scotopic contrast sensitivity loss but not photopic contrast sensitivity loss. This occurred only in bead-injected eyes (Fig. 4c, d, Table 1) and the effect increased with the magnitude of ΔICP. Interestingly, even though both eyes are exposed to the same ICP, this ΔICP effect was not detected for saline-injected control eyes, suggesting that ΔICP alone may not explain contrast sensitivity changes. Along these lines, we detected no effect of experimental TLPD on contrast sensitivity, quite similar to our observations for experimental IOP, ΔIOP, and experimental ICP (Fig. 4e, Table 1). However, for ΔTLPD, bead-injected eyes under scotopic conditions showed small losses with a ΔTLPD near zero (the TLPD was minimally deviated from baseline levels by the experiment), but very large losses at larger negative values (Fig. 4f, Table 1). This suggested that maintenance of the normal TLPD was important to preserve visual function, whereas negative deviation from the normal TLPD led to profound loss of visual function. Furthermore, this range of effect across the magnitude of TLPD only for scotopic conditions may explain why no overall effect on scotopic contrast sensitivity was detected when eyes were analyzed as a single group (Fig. 3). To explore the significant relationships between ΔICP and ΔTLPD and scotopic contrast sensitivity loss in bead-injected eyes in more detail, we performed a logistic regression between either ΔICP and ΔTLPD and the probability of scotopic contrast sensitivity loss. Both variables successfully fit the data for contrast sensitivity loss in a nonlinear manner, showing a low probability of contrast sensitivity loss with small experimental deviations from baseline values, and a high probability of contrast sensitivity loss with large experimental deviations from baseline values (Fig. 4g, h). This suggests that accounting for the magnitude of change in TLPD from baseline is important when assessing the impact of ICP elevation on vision.

**Table 1:** *P* values and *r*^2^ values for experimental conditions and contrast sensitivity loss.

Condition	Saline scotopic	Saline photopic	Bead scotopic	Bead photopic
Exp. IOP	*p* = 0.46	*p* = 0.13	*p* = 0.13	*p* = 0.97
	*r*^2^ = 0.070	*r*^2^ = 0.27	*r*^2^ = 0.26	*r*^2^ = 0.00015
ΔIOP	*p* = 0.25	*p* = 0.83	*p* = 0.59	*p* = 0.87
	*r*^2^ = 0.16	*r*^2^ = 0.0062	*r*^2^ = 0.038	*r*^2^ = 0.004
Exp. ICP	*p* = 0.48	*p* = 0.35	*p* = 0.31	*p* = 0.75
	*r*^2^ = 0.064	*r*^2^ = 0.11	*r*^2^ = 0.13	*r*^2^ = 0.013
ΔICP	*p* = 0.89	*p* = 0.44	***p*** = **0.0052**	*p* = 0.42
	*r*^2^ = 0.0027	*r*^2^ = 0.076	*r*^2^ = 0.64	*r*^2^ = 0.083
Exp. TLPD	*p* = 0.78	*p* = 0.084	*p* = 0.90	*p* = 0.86
	*r*^2^ = 0.010	*r*^2^ = 0.33	*r*^2^ = 0.0019	*r*^2^ = 0.0040
ΔTLPD	*p* = 0.63	*p* = 0.20	***p*** = **0.0070**	*p* = 0.30
	*r*^2^ = 0.030	*r*^2^ = 0.20	*r*^2^ = 0.62	*r*^2^ = 0.13

*p* values and *r*^2^ values for best fit lines presented in Fig. 4a–f. Statistically significant relationships are in bold.

### Changes in anatomy

**Fig. 5:**
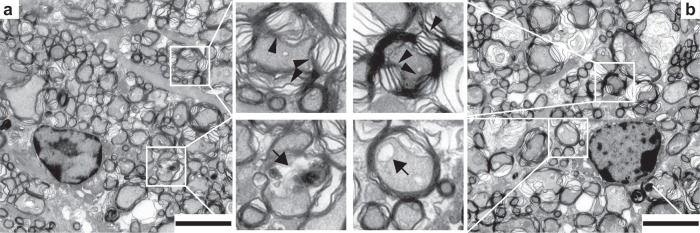
Effects of IOP and ICP elevation on optic nerve anatomy. Representative transmission electron microscope images of optic nerves collected from the bead-injected eye (**a** and inserts) and saline-injected eye (**b** and inserts) of the same animal. Qualitative assessments of paired eyes were similar, showing disruption of the myelin sheath (top inserts, arrowheads) and vacuolar degeneration of the axoplasm (bottom inserts, arrows). Scale bar = 4 µm.

We extended these analyses to cell counts from whole-mounted retinas stained for both RGC (RBPMS, Tuj1) and nuclear (TO-PRO3) markers (Table 2). We found equivalent cell counts between saline- and bead-injected eyes in both central and peripheral positions of the retina. Interestingly, the density of RBPMS cells was less than previously reported following ICP elevation, whereas the density of Tuj1 cells was higher than previously reported following ICP elevation, suggesting a potentially complex relationship among IOP, ICP, and RGC and inner retinal cell loss^34^. Next, we assessed the optic nerve. Similarly, we found no difference in axon counts between saline- and bead-injected eyes (Table 3). These counts were higher than previously reported following ICP elevation, but lower than reported for sham studies to control for ICP elevation^34^. Qualitatively, optic nerves from both saline- and bead-injected eyes displayed common features of dysfunction and death (Fig. 5), in a manner similar to previous reports^33,34^. Thus, while the processes of bead and saline injection may modify slightly the retinal and optic nerve anatomy of ICP-related phenotypes, there did not appear to be any anatomic differences between eyes as a consequence of IOP elevation.

**Table 2:** Cell counts in whole mount retinas.

	Central			Peripheral		
	Saline	Bead	*p* value	Saline	Bead	*p* value
RBPMS	1924 ± 250	1840 ± 301	0.75	1728 ± 238	1803 ± 247	0.65
Tuj1	4700 ± 298	4847 ± 232	0.72	4396 ± 265	4497 ± 187	0.65
TO-PRO3	5405 ± 344	5546 ± 281	0.77	5025 ± 314	5212 ± 219	0.42

Values are expressed as mean RGCs per mm^2^ ± 1 SEM. *N* = 10.

*Tuj1* anti-beta III tubulin, *RBPMS* RNA binding protein with multiple splicing, *TO-PRO3* nuclear stain.

**Table 3:** Optic nerve axon counts.

	Saline	Bead	*p* value
Estimated axon count per optic nerve	37,983 ± 2100	42,003 ± 4560	0.47

Estimated total axons per optic nerve ± 1 SEM. *N* = 8.

## Discussion

In this manuscript, we modeled SANS by experimentally elevating ICP in mice, and then explored unilateral IOP elevation as a potential countermeasure. We identified several key findings. First, eyes with elevated IOP showed additional losses of photopic contrast sensitivity when compared to control eyes, and these losses were essentially uniform across the entire range of change in TLPD. Second, elevated IOP impacted scotopic contrast sensitivity in a nonlinear manner, such that maintenance of the normal TLPD (small ΔTLPD) was more likely to preserve scotopic contrast sensitivity, whereas deviation from the normal TLPD (large ΔTLPD) was more likely to result in markedly abnormal scotopic contrast sensitivity. Third, these effects on vision appear to be unrelated to retinal cell body and axonal losses, which occurred similarly in all eyes exposed to ICP elevation, regardless of IOP level.

How can we explain these relationships? Since the average IOP elevation in this study was about 25% of the average ICP elevation, only a partial countermeasure was achieved. This was primarily because the method of IOP elevation^37^, which typically does not cause dramatic increases, could not match the ICP elevations needed to model SANS. However, this partial countermeasure still yielded valuable information about the nature of ICP−IOP balance because of eye to eye variability—the degree of countermeasure varied among eyes according to ΔTLPD. A larger ΔTLPD represented a partial countermeasure, whereas a smaller ΔTLPD represented a more complete countermeasure (closer to 100%). In the case of RGC and optic nerve anatomy, the ineffectiveness of IOP to prevent damage from ICP may simply reflect this partial countermeasure—the damage caused by ICP elevation could not be overcome by IOP elevation of any lesser degree. In the case of photopic contrast sensitivity, the partial countermeasure actually worsened vision loss. In the case of scotopic contrast sensitivity, the partial countermeasure showed a range of outcomes, and was effective at preserving vision in cases where the countermeasure was more complete.

One potential reason that scotopic contrast sensitivity could be preserved is that injured RGCs and their axons may function differently based on a range of IOP, ICP, and TLPD. If this is the case, then it is possible that the surviving RGCs and their axons maintained relatively normal function even while surrounding RGCs and their axons were dying, and that this normal function was facilitated by maintenance of the normal TLPD. Previous work has found that scotopic contrast sensitivity is very sensitive to IOP level, whereas photopic contrast sensitivity was sensitive to a combination of factors, including IOP level and RGC count^43^. Furthermore, in anesthetized rats, the increase of ICP in the setting of even massively elevated IOP was sufficient to prevent RGC electrical dysfunction as measured via the scotopic threshold response of the electroretinograms^18,45^. Taken together, this may suggest the scotopic pathways are most susceptible to the balance between IOP and ICP, and therefore more likely to be preserved by maintenance of the normal TLPD, even when both IOP and ICP are increased. Unlike scotopic sensitivity, photopic contrast sensitivity changes may reflect the additive effects of IOP and ICP.

On a molecular level, since both elevated IOP and ICP can impact axoplasmic flow at the optic nerve head, it is possible that these opposing pressures offset and allow for more normal energy metabolism and transport among surviving neurons^46–48^. Another possibility is that IOP elevation by bead injection induces transcriptional changes in RGCs that offer some visual preservation in the setting of a second (ICP) insult. Indeed, IOP level and duration of exposure to IOP elevation have an important impact on both RGC physiology and gene expression, and these may underlie complex relationships that determine how an RGC and its axon respond physiologically to simultaneous ICP and IOP increase^39,42,49^. Finally, several other studies have also identified a relative uncoupling of RGC anatomy and function, especially in glaucoma models^50–52^. This further suggests that the processes underlying cell death and visual physiology associated with ICP and IOP increases may simultaneously be regulated by different mechanisms that are lighting dependent. Further studies at an even wider range of ICP, IOP, and TLPD values, and especially while maintaining physiologic TLPD, might shed additional light on this topic.

We have previously reported on contrast sensitivity losses following either IOP or ICP elevation with the same experimental models^34,43^. In this manuscript, the magnitude of contrast sensitivity loss seen after both IOP and ICP elevation is about the same as seen previously after ICP elevation, but less than previously seen after IOP elevation. The pattern of contrast sensitivity loss, however, was not linear under scotopic conditions, which complicates comparison with other studies. We are therefore careful to compare our results only to internal controls to try to reduce the potential unseen effects of co-manipulation of both ICP and IOP. Nevertheless, one potential conclusion is that ICP elevation blocks some of the effects of IOP elevation on contrast sensitivity, which would hint at a mechanical mechanism of contrast sensitivity loss and preservation. The relationship is likely to be more complex, however, and the assessment of contrast sensitivity from individual retinal ganglion cells across a range of IOP, ICP, and TLPD values as described may be insightful.

The mouse optic nerve head is different from that of humans and nonhuman primates in that it lacks a collagenous lamina cribrosa and instead has an analogous structure which is generated by astrocytes^44^. Furthermore, the mouse optic nerve head derives its blood supply from the central retinal artery, without choroidal contribution^53^. Despite these differences in anatomy, it appears that at least some of the relationships predicted by the concomitant rise of IOP and ICP are preserved in mice. This is an important finding that supports the continued use of mice and other model systems to better understand this relationship which is critical to ocular health and a major contributor to blindness in diseases such as SANS and glaucoma. However, it is also important to recognize the limitations of model systems. For example, choroidal expansion influences optic nerve head biomechanics^54^ and peripapillary choroid thickness increases during spaceflight^55^, suggesting that it may influence SANS. It will therefore be important to confirm critical studies of mouse phenotypes in model systems which are more similar to humans (such as nonhuman primates), or in human ground-based analogs of weightlessness^56^.

Finally, the distinctions identified in this manuscript, especially regarding the exquisite sensitivity of scotopic contrast sensitivity to changes in the TLPD, may be worthy of consideration when assessing visual changes in humans exposed to prolonged microgravity. For example, it is possible that scotopic or dim light level visual tasks will be more rapidly impacted, thereby serving as an early indicator of SANS. Furthermore, the additive results of ICP and IOP on vision loss at certain ranges of TLPD change raise the concern that simply raising IOP to combat the effects of ICP in SANS may be too simple of an approach, potentially leading to worsening rather than amelioration of visual symptoms. This concern may be most valid in cases where a partial and not complete countermeasure is achieved.

## Methods

### Animals

Experiments were conducted in accordance with all relevant federal ethics guidelines and additionally with the ARVO Statement of the Use of Animals in Ophthalmic and Vision Research. All protocols, procedures, and ethical considerations were approved in advance by the Institutional Animal Care and Use Committee (IACUC) of Baylor College of Medicine. Ten C57BL6J mice of both genders aged 12 weeks were either procured from Jackson Labs (strain 000664) or bred from the same animal line. Animals were kept under a standard 12-h light and dark cycle with a light level of 0.10 foot candles. Mice were housed communally in cages of four animals once weaned until ICP probes were implanted, after which the animals were placed in solitary custom housing approved by the IACUC. The custom housing system provided food and enrichment on the cage floor, and water from an easily accessible position on the side of the cage.

### ICP surgery and elevation

Animals were fitted with an artificial CSF (aCSF) infusion and ICP monitoring probe as previously described with some modifications due to changes in IACUC requirements^33,34^. Briefly, animals were weighed and anesthetized with intraperitoneal rodent combination anesthetic (ketamine 80 mg/kg, xylazine 16 mg/kg, and acepromazine 1.2 mg/kg), and local anesthetic at the surgical site (0.1% lidocaine and 0.025% bupivacaine). Pain control was further achieved with meloxicam (5 mg/kg) at the time of surgery and then every 24 h for 3 days. The top of the head and the back were shaved and treated with depilatory cream before surgery. The skull was exposed and two holes of 1.2 mm diameter were drilled 1 mm posterior and 1 mm lateral to bregma. The dura was nicked with a 30-gauge needle to expose the subarachnoid space and one hole fitted with a cannula composed of a nylon screw (C212SGN, P1 Technologies, Roanoke, VA) with an inserted 22-gauge needle and the other with a stainless steel screw with a 0.5 mm central channel (C212SG, P1 Technologies). These modifications were held in place either with Durelon Carboxylate Luting Cement (Ref#:3,38234M, St. Paul, MN) or C&B Metabond Adhesive luting cement (SKU# S380, Parkell, Edgewood, NY). A pressure monitoring transmitter (PA-C10, Data Sciences International, DSI, St. Paul, MN) was placed subdermally under the back skin of the mouse, with the probe tip placed into the subarachnoid space and held in place by 6-0 sutures and silicone caulk (GE, Louisville, KY). The infusion cannula was connected with polyurethane tubing (VAHBPU-T22, OD 1.44 mm, ID 0.63 mm, Instech, Plymouth Meeting, PA) with one end sealed to close the brain cavity. Mice were allowed to recover overnight. Baseline ICP was measured daily starting at post-operative day 1 until IOP was elevated, starting the experiment (see below and Fig. 1). Intracranial pressure data were collected from the implanted probes with the PhysioTel Small Animal Telemetry system for about 1 h each day during the week and data were processed using Ponemah Software 6.11 software (DSI).

Intracranial pressure was elevated the day after IOP elevation by infusing the subarachnoid space through the infusion cannula with sterile filtered aCSF (124 mM NaCl, 2.5 mM KCl, 2.0 mM MgSO_4_, 1.25 mM KH_2_PO_4_, 26 mM NaHCO_3_, 10 mM glucose, 4 mM sucrose, 2.5 mM CaCl_2_) which was produced in house. Continuous infusion was created by filling a sterile 30 ml bottle with aCSF, suspending it above the animal’s housing enclosure, and connecting it to the infusion cannula via the same polyurethane tubing as above. The height of the bottle was positioned 30 cm above the animal’s head and then adjusted in real time to a target ICP of 10–15 mmHg above baseline. This arrangement allowed for full range of motion and mobility of the mouse within its individual enclosure, and was maintained for the entire duration of the 2-week experiment.

### IOP elevation

Mouse IOP was elevated after placement of the ICP monitor probe as previously described^37^. Briefly, mice were anesthetized with the same agents as above and eyes were dilated and anesthetized with single drops of 1% tropicamide, 2.5% of phenylephrine hydrochloride, and 0.5% proparacaine hydrochloride. A 30-gauge needle was used to perforate the cornea to insert a pulled glass micropipette of 150-μm diameter connected to a Hamilton syringe into the anterior chamber. 1.5 μL of a mixture of 6-μm (Cat# 15715-5) and 1-μm (Cat# 15713-15) diameter polystyrene microbeads (Polysciences, Inc., Warrington, PA, USA) was injected into one eye, followed by 3 μL of sodium hyaluronate (#8065183085, Provisc; Alcon Laboratories, Fort Worth, TX, USA) to seal the cornea and force the beads into the iridocorneal angle. The other eye of the same animal was given a similar treatment but with medical grade phosphate-buffered saline (PBS) replacing the bead mixture only (sodium hyaluronate was still injected) to act as a sham for IOP elevation.

### ICP and IOP measurements

Intracranial pressure was recorded daily each weekday for 1 h within the period of 9:00 AM−12:00 PM using the DSI system. The daily value was taken as the average value of ICP during the hour period. Intraocular pressure was measured twice a week using a rebound tonometer (iCare, TONOLAB, Vantaa, Finland) within the hours of 10:00 AM−2:00 PM, and represents the average of six measurements. Baseline values were obtained the day of ICP probe implant surgery and then measured twice weekly starting the day after IOP elevation. Intracranial pressure probes were calibrated to ambient pressure prior to each use.

### Measurement of contrast sensitivity

Baseline optokinetic responses (OKRs) were measured bilaterally and simultaneously on a custom system. In all cases, animals were dark adapted for at least 2 h before contrast sensitivity was measured under photopic and scotopic conditions by a trained observer using a two alternative forced choice system as previously described^43,57^. Baseline testing occurred after animal recovery from ICP probe placement and prior to IOP or ICP elevation. Repeat measurements were obtained on the final day of the 2-week experiment. The difference in log contrast sensitivity over time was calculated for each eye and used for comparison within and between eyes.

### Immunofluorescence

Mice were killed with a lethal dose of rodent combo anesthetic and their eyes harvested. Retinas were dissected, fixed in 4% paraformaldehyde, flat mounted, and blocked in 10% donkey serum. Retinas were stained with primary antibodies against mouse anti-beta-III-tubulin (Tuj1; 1:500, Biolegend, Emeryville, CA) and rabbit anti-RNA binding protein with multiple splicing (RBPMS; 1:250, PhosphoSolutions, Aurora, CO) in 3% donkey serum for 3 days at 4 °C. Secondary antibodies used were Alexa Fluor488 conjugated donkey anti-mouse IgG (1:300, Molecular Probes, Eugene, OR), Cy3 conjugated donkey anti-rabbit IgG (1:300, Jackson Lab, West Grove PA) and TO-PRO3 Iodine (Molecular Probes).

Retinas were visualized with four stitched images taken at a ×63 objective on a laser confocal microscope (LSM 800; Carl Zeiss, Oberkochen, Germany) at eight sites across the retina surface, four close to the optic nerve head (central) and four close to the periphery of the retina (periphery) according to an established protocol^37^. Counting of each of the three cellular stains was used to estimate the total number of RGCs (RBPMS) and cells overall in the RGC layer (Tuj1 and TO-PRO3) and performed in a masked manner. Total cell density was normalized to image size.

### Electron microscopy and axon counting

Optic nerves were isolated, fixed, and processed and optic nerve images obtained with the same system as previously used (Zeiss EM902 transmission electron microscope)^33,34^. Four to five regions from the central portion of the axon, along with 4−5 regions equidistant to the center and circumference of the nerve, were viewed at ×3000 magnification and imaged with a digital camera (AMT V602, Advanced Microscopy Techniques, Corp., Woburn, MA). Images were masked and myelinated axon walls identified and quantified manually from at least ten images per optic nerve. The axon density and area of the regions sampled were used to estimate axon count per optic nerve. A technical error occurred during the processing of the nerves from two animals, such that *N* = 8 for Table 2.

### Definition of key terms

Baseline values of IOP and ICP were measured prior to any elevation of pressure, and used to calculate the baseline TLPD (IOP−ICP). Experimental values of IOP and ICP were determined by averaging their post-injection values across the entire 2-week study. Experimental TLPD was determined by calculating the TLPD (IOP−ICP) at each IOP measurement point and then averaging the calculated values across the study. The difference between experimental and baseline values (experimental−baseline) was used to calculate the change (Δ) in value for each parameter, defined as ΔIOP, ΔICP, and ΔTLPD.

### Statistical methods

A paired Student’s *t* test (two-sided), two-way ANOVA, or ANOVA with repeated measures was used for the comparison of different measurements (IOP, ICP, TLPD, contrast sensitivity, cell counts, and axon counts) between bead-injected eyes and saline-injected eyes, or for the comparison of pre- and post-experiment values, as indicated. Linear regression was used to estimate the impact of experimental IOP, experimental ICP, experimental TLPD, ΔIOP, ΔICP, and ΔTLPD on contrast sensitivity loss and the *p* value of best-fit line was calculated with GraphPad Prism 6.0 (GraphPad Software, San Diego, CA). A logistic regression model was used to assess the probability of significant end-point vision loss (MATLAB version 9.4.0.813654, built-in functions *glmfit* and *glmval*). Each of the 20 eyes was represented by a binary value (1 or 0) indicating whether its post-treatment contrast sensitivity is significantly decreased (worse by 2 SD or more) compared to the average of pooled pre-treatment contrast sensitivity (average baseline vision). This set of binary data was binned into groups according to ΔICP or ΔTLPD. The probability of significant vision loss in each group was calculated and the resultant probability vs. pressure change function was fit with the following equation:

1 $$p = \frac{1}{{1 + e^{ - \left( {b_0 + b_1x} \right)}}},$$

where *x* is ΔICP or ΔTLPD, and *p* is the probability of significantly decreased contrast sensitivity. Data are presented throughout as mean ± SEM.

### Reporting summary

Further information on experimental design is available in the Nature Research Reporting Summary linked to this article.

## Supplementary information

Reporting Summary

## Data Availability

The authors declare that the data that support the findings of this study are available within the paper.
