# Supplementary material for: Modeling a potential SANS countermeasure by experimental manipulation of the translaminar pressure difference in mice
Source: NPJ Microgravity. 2020 Jul 31;6:19. doi: 10.1038/s41526-020-00109-5 (PMC7395713; doi:10.1038/s41526-020-00109-5)
Supplement: Supplementary file 1 — Reporting Summary [file 41526_2020_109_MOESM1_ESM.pdf]

nature research

Corresponding author(s): Benjamin J. Frankfort

Last updated by author(s): Jul 2, 2020

Reporting Summary

Nature Research wishes to improve the reproducibility of the work that we publish. This form provides structure for consistency and transparency in reporting. For further information on Nature Research policies, see our Editorial Policies and the Editorial Policy Checklist.

Statistics

For all statistical analyses, confirm that the following items are present in the figure legend, table legend, main text, or Methods section.

n/a	Confirmed	
☐	☒	The exact sample size (*n*) for each experimental group/condition, given as a discrete number and unit of measurement
☐	☒	A statement on whether measurements were taken from distinct samples or whether the same sample was measured repeatedly
☐	☒	The statistical test(s) used AND whether they are one- or two-sided<br>*Only common tests should be described solely by name; describe more complex techniques in the Methods section.*
☒	☐	A description of all covariates tested
☒	☐	A description of any assumptions or corrections, such as tests of normality and adjustment for multiple comparisons
☐	☒	A full description of the statistical parameters including central tendency (e.g. means) or other basic estimates (e.g. regression coefficient) AND variation (e.g. standard deviation) or associated estimates of uncertainty (e.g. confidence intervals)
☐	☒	For null hypothesis testing, the test statistic (e.g. *F*, *t*, *r*) with confidence intervals, effect sizes, degrees of freedom and *P* value noted<br>*Give P values as exact values whenever suitable.*
☒	☐	For Bayesian analysis, information on the choice of priors and Markov chain Monte Carlo settings
☒	☐	For hierarchical and complex designs, identification of the appropriate level for tests and full reporting of outcomes
☒	☐	Estimates of effect sizes (e.g. Cohen's *d*, Pearson's *r*), indicating how they were calculated

Our web collection on statistics for biologists contains articles on many of the points above.

Software and code

Policy information about availability of computer code

Data collection	Ponemah Software 6.11, Microsoft Excel
Data analysis	GraphPad Prism 6.0, Matlab Version 9.4.0.813654 (R2018a)

For manuscripts utilizing custom algorithms or software that are central to the research but not yet described in published literature, software must be made available to editors and reviewers. We strongly encourage code deposition in a community repository (e.g. GitHub). See the Nature Research guidelines for submitting code & software for further information.

Data

Policy information about availability of data

All manuscripts must include a data availability statement. This statement should provide the following information, where applicable:

- Accession codes, unique identifiers, or web links for publicly available datasets
- A list of figures that have associated raw data
- A description of any restrictions on data availability

The authors declare that the data that support the findings of this study are available within the paper.

Field-specific reporting

Life sciences study design

All studies must disclose on these points even when the disclosure is negative.

Sample size	The sample size needed was determined based on the expected level of ICP and IOP elevation according to previous studies.
Data exclusions	None
Replication	IOP and ICP are reported in all cases as the average of multiple measurements.
Randomization	These are internally controlled experiments. The eye to receive IOP elevation was randomized by coin flip with each animal.
Blinding	In all cases where possible, data was masked so that the person doing analysis would not know if an eye was in the experimental or control group.

Reporting for specific materials, systems and methods

We require information from authors about some types of materials, experimental systems and methods used in many studies. Here, indicate whether each material, system or method listed is relevant to your study. If you are not sure if a list item applies to your research, read the appropriate section before selecting a response.

Materials & experimental systems

n/a	Involved in the study	
☐	☒	Antibodies
☒	☐	Eukaryotic cell lines
☒	☐	Palaeontology and archaeology
☐	☒	Animals and other organisms
☒	☐	Human research participants
☒	☐	Clinical data
☒	☐	Dual use research of concern

Methods

n/a	Involved in the study	
☒	☐	ChIP-seq
☒	☐	Flow cytometry
☒	☐	MRI-based neuroimaging

Antibodies

Antibodies used	For primary antibodies:<br>Anti-RBPMS antibody, Phosphosolutions, catalog# 1830-RBPMS, lot# NB717p, 1:250;<br>Anti-Tubulin β 3 (TUBB3) Antibody, Biolegend, catalog#801202, lot# B205807, 1:500;<br>For secondary antibodies:<br>Alexa Fluor 488 AffiniPure Donkey-anti-Mouse IgG, catalog# 715-545-150; 1:300;<br>Cy3 AffiniPure Donkey-anti-Rabbit IgG, catalog# 711-165-152, 1:300
Validation	The primary antibodies were tested for optimal condition before the experiment. For more information about the validation, please refer to the manufacturer's websites:<br>https://www.phosphosolutions.com/shop/rbpms-antibody-rabbit/<br>https://www.biolegend.com/en-us/products/purified-anti-tubulin-beta-3-tubb3-antibody-11580

Animals and other organisms

Policy information about studies involving animals; ARRIVE guidelines recommended for reporting animal research

Laboratory animals	Mouse, C57BL6J, 12 weeks, male + female
Wild animals	None
Field-collected samples	None
Ethics oversight	Baylor College of Medicine IACUC.

Note that full information on the approval of the study protocol must also be provided in the manuscript.
